# Opium consumption and long-term outcomes of CABG surgery in patients without modifiable risk factors

**DOI:** 10.3389/fsurg.2023.1047807

**Published:** 2023-02-17

**Authors:** Ali Sheikhy, Aida Fallahzadeh, Sepehr Nayebirad, Mahdi Nalini, Saeed Sadeghian, Mina Pashang, Mahmoud Shirzad, Abbas Salehi-Omran, Soheil Mansourian, Jamshid Bagheri, Kaveh Hosseini

**Affiliations:** ^1^ Tehran Heart Center, Cardiovascular Diseases Research Institute, Tehran University of Medical Sciences, Tehran, Iran; ^2^ Cardiac Primary Prevention Research Center, Cardiovascular Diseases Research Institute, Tehran University of Medical Sciences, Tehran, Iran; ^3^ Non-Communicable Disease Research Center, Endocrinology and Metabolism Population Sciences Institute, Tehran University of Medical Sciences, Tehran, Iran; ^4^ Digestive Oncology Research Center, Digestive Diseases Research Institute, Tehran University of Medical Sciences, Shariati Hospital, Tehran, Iran; ^5^ Cardiovascular Research Center, Kermanshah University of Medical Sciences, Imam Ali Hospital, Shahid Beheshti Boulevard, Kermanshah, Iran

**Keywords:** coronary artery bypass grafting, coronary artery disease, opium, heart, smurf

## Abstract

**Background:**

The question about the significance of opium consumption as a coronary artery disease (CAD) risk factor still remains open. The present study aimed to evaluate the association between opium consumption and long term outcomes of coronary artery bypass grafting (CABG) in patients without **s**tandard **m**odifiable CAD **r**isk **f**actors (SMuRFs; hypertension, diabetes, dyslipidemia, and smoking).

**Methods:**

In this registry-based design, we included 23,688 patients with CAD who underwent isolated CABG between January 2006 to December 2016. Outcomes were compared in two groups; with and without SMuRF. The main outcomes were all-cause mortality, fatal and nonfatal cerebrovascular events (MACCE). Inverse probability weighting (IPW) adjusted Cox's proportional hazards (PH) model was used to evaluate the effect of opium on post-op outcomes.

**Results:**

During 133,593 person-years of follow-up, opium consumption was associated with increased risk of mortality in both patients with and without SMuRFs (weighted Hazard Ratio (HR)s: 1.248 [1.009, 1.574] and 1.410 [1.008, 2.038], respectively). There was no association between opium consumption and fatal and non-fatal MACCE in patients without SMuRF (HR = 1.027 [0.762–1.383], HR 0.700 [0.438–1.118]). Opium consumption was associated with earlier age of CABG in both groups; 2.77 (1.68, 3.85) years in SMuRF-less and 1.70 (1.11, 2.38) years in patients with SMuRFs.

**Conclusion:**

Opium users not only undergo CABG at younger ages but also have a higher rate of mortality regardless of the presence of traditional CAD risk factors. Conversely, the risk of MACCE is only higher in patients with at least one modifiable CAD risk factor.

## Introduction

The standard modifiable cardiovascular risk factors (SMuRFs), which are diabetes mellitus (DM), dyslipidemia (DLP), hypertension (HTN) and cigarette smoking (CS), are the key elements of the Framingham risk score (1) and are targeted in primary and secondary prevention programs (2). However, an increasing number of patients with established coronary artery disease present with no known SMuRFs (SMuRF-less patients) at the time of first diagnosis (3, 4). Several large registry-based studies have compared the short- and long-term outcomes with their counterparts with at least one SMuRF (3, 5). However, the results were conflicting and some studies reported a higher risk of mortality in SMuRF-less patients while some reported no significant differences between patients with and without SMuRFS. Although outcomes of STEMI subjects with no SMuRFs have been widely studied, there has been hardly any studies focusing specifically on SMuRF-less patients undergoing CABG.

While the traditional SMuRFs have been used to predict the cardiovascular risk of an individual, there are other possible risk factors such as opium consumption that can affect the patients’ outcomes. Opium consumption is highly prevalent in developing countries of the Middle East and Asia, especially in Iran (6, 7). The high prevalence of opium use in Iran is partially due to the ease of access and also the misconception among the Iranian population and even medical staff that opium might decrease the risk of certain medical conditions such as diabetes, hypertension as well as CAD (8). Although many studies have reported detrimental effects of opium use on the cardiovascular system and poor post CABG outcomes in opium users (8–10), it is still not clear whether opium consumption should be considered as an independent CAD risk factor (besides other SMuRFs) or not. In addition, the effect of opium on long-term outcomes of CABG in SMuRF-less patients is debatable. Hence, in the present study, we aimed to evaluate the association of opium use with outcomes of isolated CABG in the SMuRF-less group and compare it with patients with at least one SMuRF.

## Material and method

### Study population, setting, and design

In this registry-based retrospective cohort study, which performed at Tehran Heart Center (THC) (11) from January 2006 to December 2016, patients undergoing isolated CABG surgery were included; all pre-operative and intraoperative data were gathered from the health information system (HIS). Postoperative and follow-up data were collected prospectively. We reported this study according to the Strengthening the Reporting of Observational Studies in Epidemiology (STROBE) statement. Patients with inadequate data were excluded from the current study. There were two inclusion criteria; (1) Surgical revascularization criteria for ischemic heart disease, and (2) Isolated CABG excluding valve surgeries. Ultimately, 23,688 patients were included in the final analysis. The SMuRFs assessed in this study included HTN, DLP, DM, and current CS. Patients were divided into two main groups according to their SMuRF score (with and without SMuRFs). The present study was approved by the ethical board of THC (IR-THC-13799) and the involved human data was in accordance with the Helsinki Declaration.

### Follow-up protocol

Subjects were followed-up at 4, 6, and 12 months after surgery. After the first year of follow-up, patients were visited annually. Follow-up visits were carried out in the center's post-op clinic and data regarding mortality and MAACE were collected.

### Definition of variables

Diabetes mellitus was defined as fasting plasma glucose ≥126 mg/dl, random plasma glucose ≥200 mg/dl, hemoglobin A1c (HbA1c) ≥6.5% (12), treatment with either oral hypoglycemic agents or insulin. Hypertension was defined as a minimum systolic blood pressure of 140 mm Hg, a minimum diastolic blood pressure of 90 mm Hg or a history of antihypertensive therapy (13). Dyslipidemia was defined as the presence of a minimum total cholesterol level of 240 mg/dl, a minimum triglyceride level of 200 mg/dl, or a high-density lipoprotein cholesterol level of less than 40 mg/dl in men and less than 50 mg/dl in women or a minimum low-density lipoprotein cholesterol level of 160 mg/dl, or a history of prescribed lipid medications based on the National Cholesterol Education Program (NCEP) Adult Treatment Plan (ATP) III (14). A family history of CAD was defined as having a first-degree relative with a history of CAD including acute myocardial infarction or documented CAD (through invasive coronary angiography or computed tomography coronary angiography).' Current smoking was defined as regularly smoking more than one cigarette per day as reported by the patient. Opium consumption was defined as the current consumption of opium either smoking opium or drinking opium dissolved in tea.

### Study outcomes

The primary outcomes were defined as all-cause mortality, MACCE (major adverse cardiac and cerebrovascular events) and non-fatal MACCE (comprising of non-fatal acute coronary syndromes [ACS], non-fatal stroke or transient ischemic attack [TIA], and repeated coronary revascularization *via* percutaneous coronary intervention [PCI] or redo-CABG).

### Statistical analysis

Mean with standard deviation (SD) and median with 25th and 75th percentiles [interquartile range (IQR) boundaries] were used to present normal and skewed continuous variables, respectively. The normality of the variables was assessed using histogram charts in addition to the central tendency and dispersion measures. Comparison between “opium consumers” and “non-consumers” groups was done using student's *t*-test for normally distributed and Mann–Whitney *U*-test for skewed distributed variables. Categorical variables were expressed as frequency and percentage which were compared between the two abovementioned groups using the chi-squared test. Inverse probability weights (IPW) were used to stabilize potential selection biases of treatment, weights were calculated from propensity score (PS) **(**Supplementary Figure S1**)**, which was generated by predicted probabilities of logistic regression on identified potential confounders. All selected variable in the PS estimation model was mentioned in (Supplementary Table S1**)**. The C-statistic for the model was 0.83 (Supplementary Figure S2**)**. Weights for each case (Wi) were calculated as 1/PS(Xi) for opium consumers, and 1/[1-PS(Xi)] for non-consumers. The standardized mean difference (SMD) was used as a balance metric to evaluate the difference between distributions of a pre-treatment variable, a balance indicator considered as “SMD < 0.1” **(**Supplementary Figure S3**)**.

The weighted and unadjusted effects of opium consumption on all-cause mortality and MACCE were obtained using Cox's proportional hazards (PH) model. Interactions were examined by including appropriate interaction terms in the Cox regression models and reported as the ratio of HR (RHR) by considering SMuRF positive group as reference. Adjusted linear regression was used to assess the association between opium use with the age of CABG; hence in this model, age was considered an outcome.

All statistical analyses were conducted applying R version 4.0.3, moreover, we used several packages in R: survival” (package for survival analysis in R), “survminer” (drawing survival curves), and “ggplot2”. All *P*-values are two-sided; moreover, *P*-values <0.05 were considered statistically significant.

## Results

### Baseline characteristics

A total of 23,688 patients who underwent isolated CABG were recruited. During 133,593 person-years of follow-up (median 74.64; 25th–75th percentile: 73.97–75.32 months), barely 2% of the patients (485) were lost to follow-up. 3,432 (14.49%) patients did not have any SMuRFs (SMuRF-less). Baseline features are shown in Table 1.

**Table 1:** Baseline characteristics based on the presence of SMuRFs and opium consumption.

		SMuRF-less (*n* = 3,432)			SMuRF=>1 (*n* = 20,256)		
		Non-opium users (*n* = 3,066)	Opium users (*n* = 366)	*P* value	Non-opium users (*n* = 17,380)	Opium users (*n* = 2,876)	*P* value
Gender	Female	421 (13.7%)	2 (0.5%)	<0.001	5,808 (33.4%)	130 (4.5%)	<0.001
	Male	2,645 (86.3%)	364 (99.5%)		11,572 (66.6%)	2,746 (95.5%)	
Age (years)		62 (1)	58 (1)	<0.001	61 (1)	57 (1)	<0.001
BMI (kg/m^2^)	BMI <30	2,556 (83.3%)	298 (81.4%)	0.367	12,842 (74.2%)	2,279 (79.6%)	0.115
	BMI ≥30	510 (16.7%)	68 (18.6%)		4,468 (25.8%)	583 (20.4%)	
Hb (g/dl)		14.11 (0.08)	14.03 (0.03)	0.423	13.70 (0.01)	13.91 (0.03)	0.228
Graft number		3 (3,4)	4 (3,4)	0.201	3 (3,4)	3 (3,4)	0.049
Diabetes		NA	NA	–	8106 (46.6%)	1,008 (35.0%)	<0.001
Hypertension		NA	NA	–	11,165 (64.2%)	1,478 (51.4%)	<0.001
Dyslipidemia		NA	NA	–	11,733 (67.5%)	1,590 (55.3%)	<0.001
Current CS		NA	NA	–	2,553 (14.7%)	1,503 (52.3%)	<0.001
eGFR (ml/min)		83.45 (66.80, 102.92)	94.21 (74.68, 110.91)	<0.001	84.37 (65.98, 105.19)	93.27 (73.86, 115.37)	<0.001
Positive Family History		983 (32.1%)	131 (35.8%)	0.153	6,905 (39.8%)	1,055 (36.7%)	0.002
EF (%)	≥50	1,332 (48.3%)	145 (42.4%)	0.046	8,685 (55.0%)	1,158 (43.2%)	<0.001
	<50	1,427 (51.7%)	197 (57.6%)		7,115 (45.0%)	1,520 (56.8%)	
LM stenosis		276 (9.0%)	26 (7.1%)	0.226	1,407 (8.1%)	253 (8.8%)	0.204
Pre-Surgery PCI		154 (5.0%)	33 (9.0%)	0.002	1,117 (6.4%)	235 (8.2%)	0.001
Renal Failure		35 (1.1%)	3 (0.8%)	0.577	449 (2.6%)	73 (2.5%)	0.889
Urgent operation		122 (4.0%)	15 (4.1%)	0.924	968 (5.6%)	152 (5.3%)	0.531
COPD		81 (2.7%)	18 (5.0%)	0.013	412 (2.4%)	126 (4.4%)	<0.001
Cerebrovascular Accident		111 (3.6%)	16 (4.4%)	0.470	1,075 (6.2%)	210 (7.3%)	0.024
Pre-CABG MI Interval	No MI	2017 (65.8%)	199 (54.4%)	<0.001	11,779 (67.8%)	1,649 (57.3%)	<0.001
	≤ 7Day	220 (7.2%)	23 (6.3%)		1295 (7.5%)	314 (10.9%)	
	8–21 day	158 (5.2%)	25 (6.8%)		840 (4.8%)	218 (7.6%)	
	>21 Day	671 (21.9%)	119 (32.5%)		3,466 (19.9%)	695 (24.2%)	

Results are reported as *N*(%), Median (IQR), and Mean (SD).

BMI, body mass index; Hb, hemoglobin; CS, cigarette smoking; eGFR, estimated glomerular filtration rate; EF, ejection fraction; LM, left main; PCI, percutaneous coronary intervention; COPD, chronic obstructive pulmonary disease; MI, myocardial infarction; CABG, coronary artery bypass grafting.

In the SMuRF-less group, 366 (10.66%) patients were opium users. Age at the time of admission was significantly lower in opium users (*P* < 0.001).

In patients with at least one SMuRF, 2,876 (14.20%) were opium consumers. The average age of the patients at the time of admission was significantly lower in opium users (*P* < 0.001). In both study groups, the number of male subjects was significantly higher in the opium consumption subcategory (*P* < 0.001).

### Mortality

**Figure 1:**
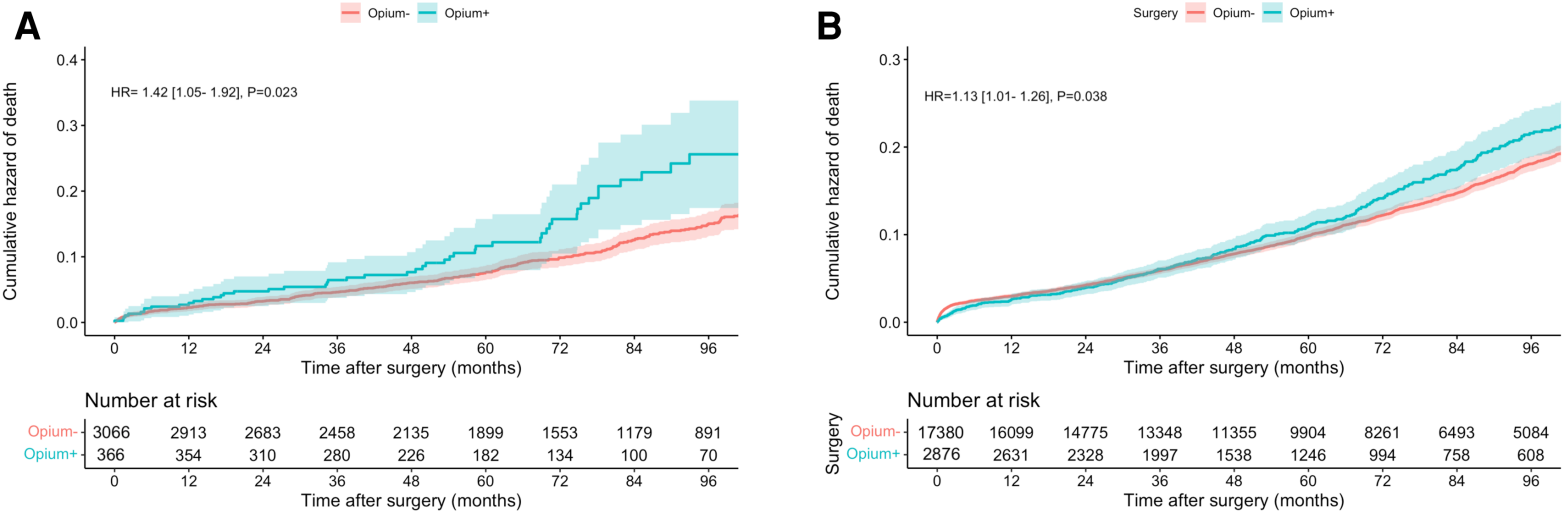
Cumulative hazard of mortality in patients without (**A**) and with (**B**) SMuRF.

At six years of follow-up, the mortality rate in entire study was 11.8%. Association between opium consumption and all-cause mortality in patients with and without SMuRFs was assessed, Table 2 and Figures 1A,B. In patients without any SMuRFs (SMuRF-less), the mortality trend was significantly different and was worse in the opium consumer group [HR:1.410 (1.008, 1.925), *P* = 0.024], Figure 1A.

**Table 2:** Main outcomes.

	SMuRF-less				SMuRF+				Interaction of SMuRF-Opium	
Main outcomes	Hazard ratio (Crude model)		Hazard ratio (IPW adjusted model)		Hazard ratio (Crude model)		Hazard ratio (IPW adjusted model)			
	HR	*P* value	HR	*P* value	HR	*P* value	HR	*P* value	RHR	*P* for interaction
Mortality	1.421 [1.049–1.925]	0.023	1.410 [1.008–2.038]	0.024	1.129 [1.009–1.262]	0.038	1.248 [1.021–1.574]	0.002	1.007 [0.595–1.405]	0.682
MACCE	1.120 [0.883–1.420]	0.351	1.027 [0.762–1.383]	0.862	1.098 [1.014–1.188]	0.021	1.201 [1.016–1.419]	0.003	0.839 [0.676–1.234]	0.312
Non-fatal MACCE	0.838 [0.577–1.217]	0.353	0.700 [0.438–1.118]	0.136	1.099 [0.986–1.226]	0.087	1.001 [0.866–1.399]	0.433	0.633 [0.995–0.387]	0.038

Subgroup analysis was done to assess all-cause mortality in each traditional CAD risk factor, Table 3. Mortality risk due to opium consumption was barely higher in the SMuRF-less group [Ratio of Hazard Ratio (RHR) = 1.007, CI: 0.595–1.405; P-interation = 0.682]. Mortality was significantly higher in non-hypertensive and non-diabetic patients who consumed opium.

**Table 3:** Mortality hazard ratios for opium consumption based on each CAD risk factor.

	Crude model		IPW adjusted model		P for interaction
	HR	*P* value	HR	*P* value	
Not cigarette smokers	1.241 [1.087–1.417]	0.001	1.240 [0.825–1.863]	0.300	0.148
Cigarette smokers	1.114 [0.922–1.346]	0.262	1.223 [0.935–1.602]	0.142	
Non diabetic	1.242 [1.086–1.421]	0.002	1.368 [1.047–1.789]	0.022	0.038
Diabetic	1.149 [0.971–1.358]	0.106	1.167 [0.842–1.618]	0.354	
Non hypertensive	1.407 [1.213–1.632]	<0.001	1.559 [1.197–2.028]	<0.001	0.002
hypertensive	1.052 [0.906–1.220]	0.508	1.088 [0.792–1.495]	0.602	
Non dyslipidemic	1.226 [1.061–1.416]	0.006	1.490 [1.188–1.869]	<0.001	0.130
Dyslipidemic	1.086 [0.932–1.265]	0.291	1.089 [0.768–1.543]	0.632	

### MACCE

**Figure 2:**
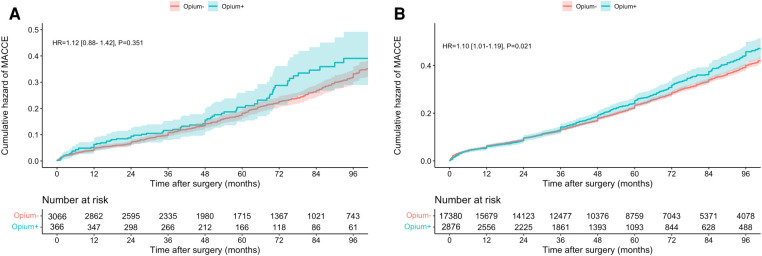
Cumulative hazard of MACCE in patients without (**A**) and with (**B**) SMuRF.

Table 2 and Figure 2 demonstratethe association between pre-operative opium consumption and long-term MACCE. Although the trend shows a lower rate of MACCE in the SMuRF-less group compared to patients with SMuRF [HR:1.027 (0.762–1.383)], it was not statistically significant during follow-up, Figure 2A (*P* = 0.862). in contrast, patients with at least one SMuRF, opium consumption was associated with a higher rate of MACCE [HR: 1.201 (1.016, 1.419)]. Similar to mortality, MACCE was almost similar in the first 3 years but became divergent afterwards, Figure 2B. Subgroup analysis did not show significant differences of MACCE in studied subgroups, Table 4. In SMuRF less population, the rate of ACS, CVA, and revascularization was higher in non-opium consumers (7.3% vs. 6.1%, 2.5% vs. 1.9%, and 1.7% vs. 0.3%, respectively); In patients with at least one SMuRF, opium consumption was significantly associated with higher ACS [HR: 1.196 (1.052–1.360), *P* = 0.006]. The number of these components of MACCE were comparably low, hence this study did not have enough power to report generalizable results.

**Table 4:** MACCE hazard ratios for opium consumption based on each CAD risk factor.

	Crude model		IPW adjusted model		P for interaction
	HR	*P* value	HR	*P* value	
Not cigarette smokers	1.086 [0.985–1.198]	0.096	1.289 [1.012–1.708]	0.001	0.823
Cigarette smokers	1.153 [1.011–1.314]	0.034	1.253 [1.037–1.514]	0.019	
Non diabetic	1.140 [1.037–1.254]	0.007	1.153 [1.023–1.399]	0.002	0.605
Diabetic	1.127 [0.996–1.275]	0.057	1.213 [0.957–1.537]	0.061	
Non hypertensive	1.168 [1.049–1.301]	0.005	1.284 [1.060–1.557]	0.011	0.432
hypertensive	1.109 [0.998–1.232]	0.055	1.106 [0.877–1.394]	0.396	
Non dyslipidemic	1.147 [1.029–1.279]	0.014	1.252 [1.062–1.475]	<0.001	0.221
Dyslipidemic	1.069 [0.963–1.186]	0.210	1.122 [0.882–1.426]	0.347	

### Non-fatal MACCE

There was no association between opium consumption and non-fatal MACCE in both SMuRF and SMuRF-less groups (HR: 1.138 [0.981–1.322] *P* = 0.088 and HR: 0.856 [0.543–1.349] *P* = 0.503, respectively).

### Opium consumption and age of CABG

The average age in patients with and without SMuRFs was 56.89 (53.44, 60.33) and 62.12 (60.05, 64.19), respectively, Table 5. Opium consumers underwent CABG at an earlier age when compared with the nonusers in both SMuRF-less and SMuRF + groups. The effect, however, was more pronounced in the SMuRF-less group (2.77 years earlier, 95% CI 1.68–3.85 in the SMuRF-less group vs. 1.70, 95% CI 1.11–2.38 in SMuRF + group).

**Table 5:** Association of opium consumption with the age of CABG.

	Mean of age	Opium consumption effect^a^	*P* value
SMuRF-less	56.89 (53.44, 60.33)	−2.79 (−1.68, −3.85)	<0.001
SMuRF+	62.12 (60.05, 64.19)	−1.70 (−1.11, −2.39)	<0.001

^a^ Years reduced.

## Discussion

The present study is a large cohort of 23,688 CABG subjects highlighting the effects of opium consumption on long-term outcomes in a commonly neglected subpopulation of CAD patients, the SMuRF-less group. Our findings showed that regardless of the patients' SMuRF status, opium consumption was associated with an increased risk of long-term mortality and MACCE. Opium consumers, especially those without SMuRFs, also demonstrated susceptibility to coronary events requiring CABG at an earlier age when compared with their nonuser counterparts.

A clinically significant proportion of patients with coronary diseases have none of the traditional CAD risk factors known as SMuRFs (3). The question about better or poorer prognosis of SMuRF-less patients after CAD events is still on the table. Some studies have shown an increased all-cause mortality rate (in-hospital and 30-day mortality) in SMuRF-less patients presenting with STEMI, particularly in women (3). On the other hand, another study have shown that 5 year mortality was lower in SMuRF-less patients although it was not statistical significant after multivariate adjustment (15). This debate has also been raised about the role of opium consumption in occurrence and prognosis of CAD patients; especially more severe CAD groups who need CABG surgery.

Based on our findings, opium consumption was associated with increased risk of long-term all-cause mortality, in both patients with and without SMuRFs and also with increased risk of MACCE in patients with SMuRFs, but not with non-fatal MACCE. The possible explanation for this could be that opium consumption can mask some CAD symptoms such as chest pain; therefore, opium consumers have less hospitalization due to non-fatal MACCEs.

In accordance with the current study results, Masoudkabir et al. suggested that post-CABG opium consumption is associated with an increased risk of long-term mortality and MACCE (9).

Safaii et al. evaluated the effect of opium consumption on the short-term outcomes after CABG and showed that opium usage was associated with an increased risk of rehospitalization within six months of CABG (8). Another study conducted by Nalini et al. showed that long-term opium consumption was associated with increased risk of cardiovascular mortality, independent of traditional CAD risk factors (16).

Below we will thoroughly discuss the mechanisms in which opium may be associated with CAD occurrence and its relationship with traditional CAD risk factors will also be reviewed.

The exact mechanisms through which opium may result in increased risk of MACCE are dabating; however, some possible mechanisms have been reported based on previous studies. Studies have reported that opium may induce chronic inflammation and oxidative stress by stimulating pro-inflammatory cytokines and thus, lead to coronary atherosclerosis and occurrence of acute events (17). Another possible mechanism is that opium-addicted men and women have lower testosterone and estrogen levels than controls (14). Plasma testosterone and estrogen levels are associated with the extent of CAD and the risk of cardiovascular mortality (18, 19). In addition to traditional risk factors, studies have shown that opium consumption is associated with higher levels of several novel cardiovascular risk factors, including lipoprotein a (Lpa), c-reactive protein (CRP), fibrinogen (20), and Factor VII (21). Lpa is shown to be an indicator of premature atherosclerosis (22), CRP is an inflammatory biomarker and is associated with increased risk of CAD (23) Fibrinogen and Factor VII are also shown to be associated with CAD (24). It is probable that opium consumption causes an elevation in the inflammatory and thrombogenic biomarkers. This may explain the association between opium use and increased risk of MACCE.

### Opium and DM

Opium consumption was associated with hyperinsulinemia due to changes in hepatic extraction of insulin, hyperglycemia similar to what is seen in type 2 DM, and also high levels of glycated hemoglobin (HbA1c) and poor glycemic control (25–27). This may explain our finding that opium consumption significantly increased mortality risk in non-diabetics and confirm that the potential effect of opium usage on risk of mortality is independent of traditional CAD risk factors such as diabetes.

### Opium and HTN

long-term opium consumption may induce high blood pressure due to impact on coronary dysfunction, increase plasma homocysteine and fibrinogen levels, and consequent vascular narrowing (25). Moreover, it has been shown that opium consumption does not improve hypertension (28). According to our results, opium use was associated with increased risk of mortality in both patients with and without hypertension. However, the increase was significantly higher in patients without hypertension, possibly due to lack of screening and antihypertensive treatment in such patients.

### Opium and smoking

This study showed that opium consumption was associated with an increased risk of mortality in non-smokers. Similarly, a previous nested case-control study demonstrated that opium addiction was associated with increased risk of CAD in non-smokers; however, this association was not significant in smokers (29). This finding would emphasize that opium consumption is a risk factor for CAD, independent of cigarette smoking.

### Opium and dyslipidemia

The possible mechanisms through which opium may affect blood lipids are decreased hepatic clearance of LDL and increased hepatic synthesis of triglycerides (30). However, the studies regarding the impact of opium on lipid indices are conflicting. Several studies showed no significant association between opium addiction and lipid profile (31, 32), while some studies showed harmful effects of opium usage on lipid indices (33, 34). According to our results, opium consumption was associated with an increased mortality risk in patients without dyslipidemia, but this association was not significant in those with baseline dyslipidemia. Definition of dyslipidemia, low-density lipoprotein level, and statin use are among the main determinants of this association, which are beyond the scope of the present study.

### Opium and age of CABG

As mentioned above, opium significantly decreased the age of CABG in both SMuRF-less and SMuRF groups. This finding could be due to the possible effects of opium on the atherosclerosis by decreasing plasma testosterone (14), increasing inflammation and pro-inflammatory cytokines (17), and suppressing autonomic nervous system and thus decreasing enkephalin production in cardiomyocytes (35) which all may happen regardless of baseline CAD risk factors. Our results are in line with two other studies which evaluated the relation between opium use and age of CAD event. Roohafza et al. (36) showed that opium use is associated with younger age of myocardial infarction, which was also emphasized later in a study by Hasandokht et al. (37).

## Limitations & strength

Evaluating the cause of death during follow-up was beyond the scope of this study; hence we cannot categorize cardiac and non-cardiac death. We do not exactly know opium reduces which type of death and it needs further studies with autopsy protocols. The absolute value of blood pressure (systolic and diastolic) was not available for all patients. The amount, duration, and type of opium use may impact our results, which none of the above mentioned variables registered in our databank. We could not capture the development of hypertension or diabetes after the initial visit. Another important limitation was that we did not have data regarding the patients’ socioeconomic status, which could influence mortality regardless of opium consumption. However, implanting IPW adjustment, large sample size, noticeable follow-up duration, and documentation of cardiovascular events and mortalities are among the strength of the present study.

## Conclusion

In patients without any modifiable CAD risk factors,opium consumption was associated with higher all-cause mortality but was not associated with more MACCE and non-fatal MACCE. Opium users were younger than their counterparts which indirectly emphasizes the role of opium on earlier CAD occurrence.

## Data Availability

The datasets generated during and/or analysed during the current study are available from the corresponding author on reasonable request.
