# Supplementary material for: Opium consumption and long-term outcomes of CABG surgery in patients without modifiable risk factors
Source: Front Surg. 2023 Feb 17;10:1047807. doi: 10.3389/fsurg.2023.1047807 (PMC9982127; doi:10.3389/fsurg.2023.1047807)
Supplement: Supplementary file 1 [file Datasheet1.docx]

***Definition of variables***

DM was defined as fasting plasma glucose ≥ 126 mg/dL and/or random plasma glucose ≥ 200 mg/dL and/or hemoglobin A1c (HbA1c) ≥ 6.5% [1] and/or treatment with either oral hypoglycemic agents or insulin. HTN was defined as a minimum systolic blood pressure of 140 mm Hg or a minimum diastolic blood pressure of 90 mm Hg or a history of antihypertensive therapy [2]. DLP was defined as the presence of a minimum total cholesterol level of 240 mg/dL, a minimum triglyceride level of 200 mg/dL, or a high-density lipoprotein cholesterol level of less than 40 mg/dL in men and less than 50 mg/dL in women or a minimum low-density lipoprotein (LDL) cholesterol level of 160 mg/dL, or a history of prescribed lipid medications. A family history of CAD was defined as having a first-degree relative with a history of CAD including acute myocardial infarction or documented CAD. CS status was defined as current smoker and determined from the patient’s self-reported status. Opium consumption was defined as the current consumption of opium either smoking opium or drinking opium dissolved in tea.

1. *Standards of Medical Care in Diabetes—2014.* Diabetes Care, 2014. **37**(Supplement 1): p. S14-S80DOI: 10.2337/dc14-S014.

2. Whelton, P.K., et al., *2017 ACC/AHA/AAPA/ABC/ACPM/AGS/APhA/ASH/ASPC/NMA/PCNA Guideline for the Prevention, Detection, Evaluation, and Management of High Blood Pressure in Adults: A Report of the American College of Cardiology/American Heart Association Task Force on Clinical Practice Guidelines.* J Am Coll Cardiol, 2018. **71**(19): p. e127-e248DOI: 10.1016/j.jacc.2017.11.006.

| Supplementary table 1  **Variables used in propensity score estimations** |
| --- |
| **Demographic**  Female  Age  BMI <30 and ≥30  Current cigarette smoker |
| **Medical history**  Diabetes  Hypertension  COPD  Dyslipidemia  Cerebrovascular accident  Pre surgery PCI  Positive family history |
| **Preoperative lab test**  GFR |
| **Cardiac status**  Graft number  Ejection fraction  LM stenosis  Previous myocardial infarction (No history, ≤7 days, 8-21 days, >21days**)** |
| **Surgical decision**  Urgent operation  Off-pump Surgery |


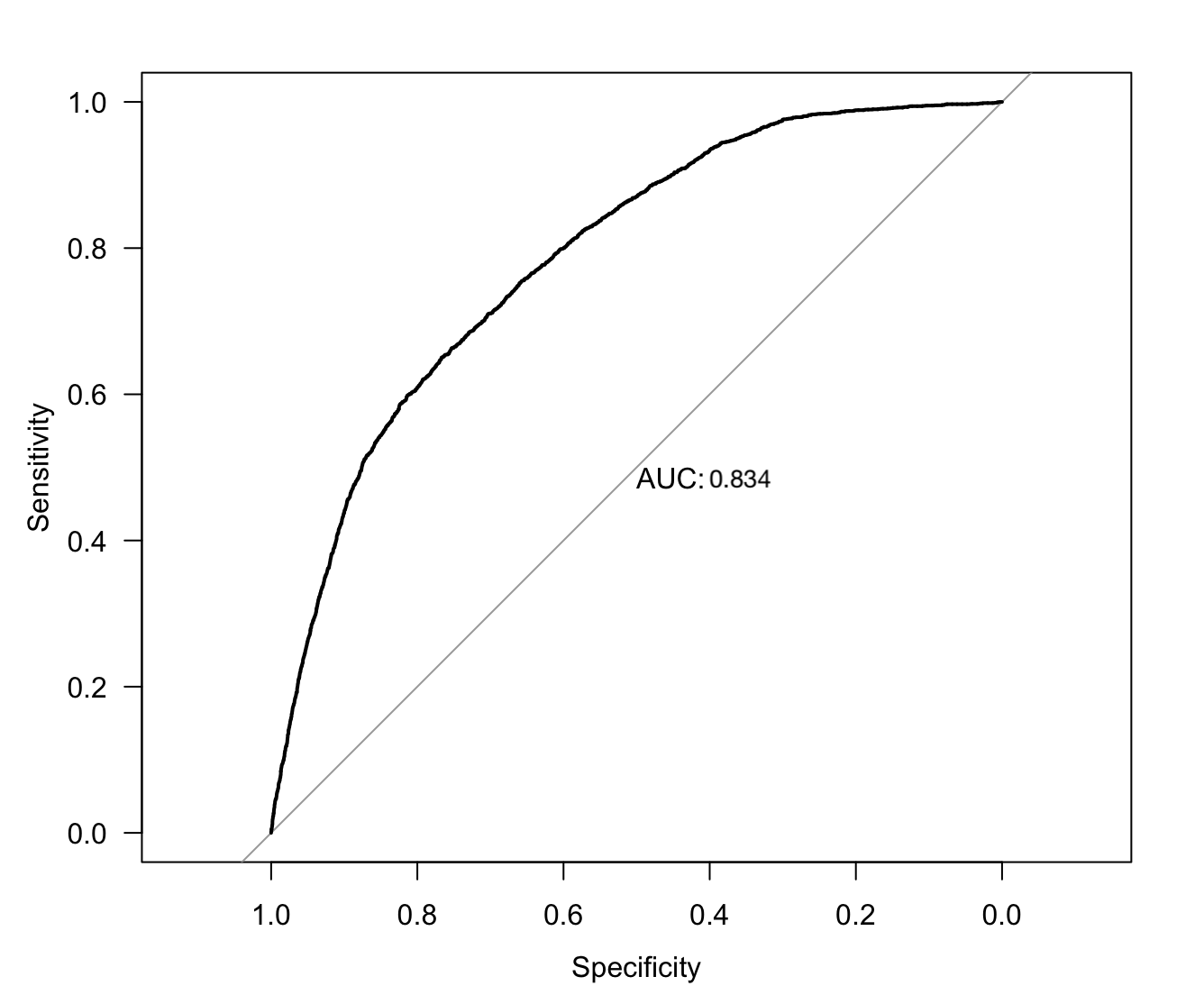


Fig1. C-statistic for propensity score modeling


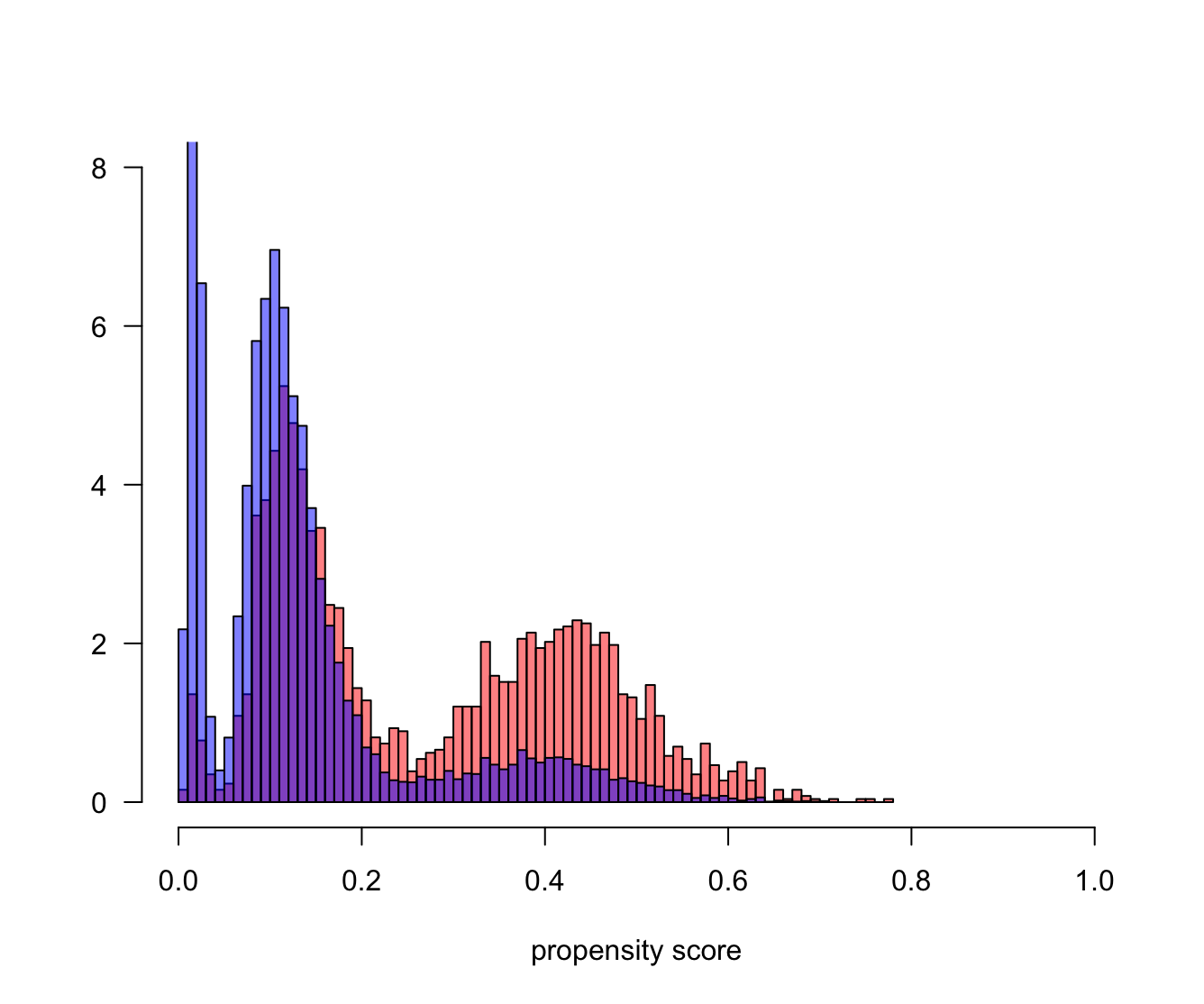


Fig 2. Estimated Propensity scores


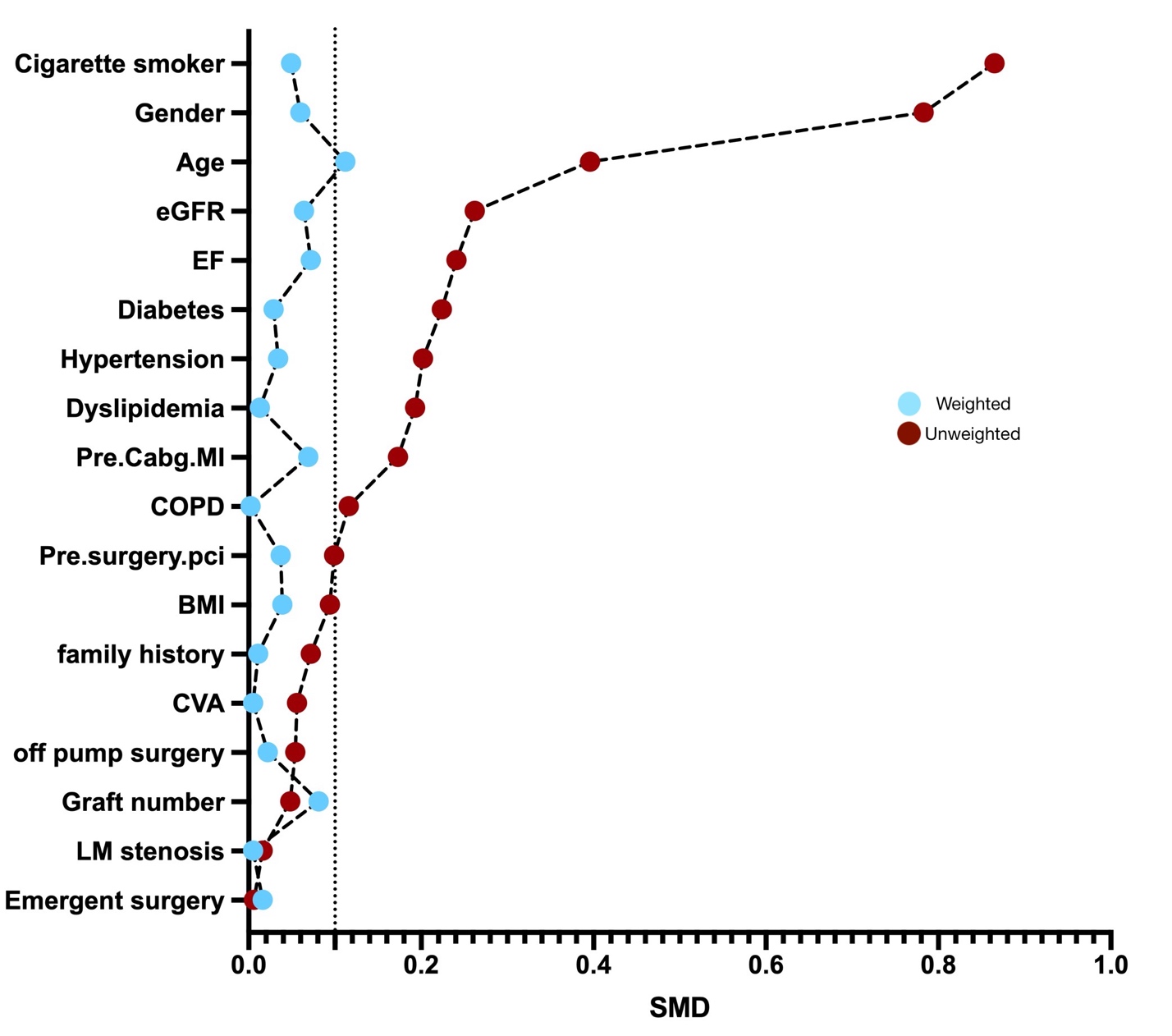


Fig 3. covariates balance estimation
